# Formulation and Evaluation of Controlled-Release Tablet of Zolpidem Tartrate by Melt Granulation Technique

**DOI:** 10.5402/2011/208394

**Published:** 2011-06-27

**Authors:** Shailesh T. Prajapati, Amit N. Patel, Chhagan N. Patel

**Affiliations:** Department of Pharmaceutics, Shri Sarvajanik Pharmacy College, Mehsana, Gujarat 384001, India

## Abstract

The present investigation describes the influence of the concentration of PEG 6000 as a melt binder and ratio of HPMC K4M : PVP on Zolpidem tartrate controlled-release tablet formulations using 3^2^ full factorial design. The ratio of HPMC K4M and PVP K30 (*X*_*1*_) and the concentration of melt binder (*X*_*2*_) were selected as independent variables, and drug release at 1 hr (*Q*_*1*_), 4 hr (*Q*_*4*_), 8 hr (*Q*_*8*_), diffusion coefficient (*n*), and release rate constant (*K*) were selected as a dependent variable. Tablets were prepared by melt granulation technique and evaluated for various evaluation parameters. It was observed that concentration of melt binder had significant effect on *Q*_*1*_, *Q*_*4*_, *n*, and *K* Binder concentration 25% w/w was found optimum. Optimized formulation (*F*_*7*_) showed good similarity with theoretical profile of drug. The *X*_*2*_ variable had a significant effect on dependent variables, and the *X*_*1*_ variable had no significant effect on dependent variables.

## 1. Introduction

Controlled-release (CR) formulations have been introduced into drug therapy with two main purposes: to reduce the number of single doses per day improving patient compliance of treatments and to decrease the fluctuations of plasma levels, in order to obtain better therapeutic efficacy and lower toxicity. There are many controlled-release pharmaceutical systems currently known, ranging from monolithic matrices, membrane reservoirs, erodible polymers, to the more technologically complex and sophisticated pH independent formulations, ion exchange resins, osmotically, and geometrically modified systems. Many of these systems are not produced in a form that is amenable to large-scale manufacturing processes and usually do not exhibit the desirable zero-order release kinetics. In addition, the cost of formulation development, raw materials, and manufacture technology are among the principal factors in CR delivery systems formulation for oral dosing [1]. An interesting approach to develop CR matrix formulations is based on melt granulation, which is a very short and easy one-step technique converting fine powders into granules. The powder agglomeration is promoted by the addition of a low melting point binder, which is solid at room temperature and melts at relatively low temperatures (50–80°C). The interest in melt granulation has increased due to the advantages of this technique over other CR delivery technologies. Since it is a solvent-free process, the drying phase is eliminated, and thus it becomes less consuming in terms of time and energy [2, 3].

Zolpidem is a nonbenzodiazepine analogue of imidazopyridine class. Zolpidem tartrate is a GABA agonist (sedative and hypnotic) used in the treatment of insomnia dosing ranging from 5 to 12.5 mg. The half life of the drug is about 1.9 to 3 hr, and oral bioavailability is 72 ± 7% indicating its promising candidature for the controlled-release formulation [4]. Zolpidem was marketed as the immediate release product in the short-term treatment of insomnia. Zolpidem is effective in reducing the time to sleep onset and increasing total sleep time; however, its effect on sleep maintenance has not been consistently demonstrated. The hypnotic effects of Zolpidem have been reported primarily in the first 3 hours postdose which can lead to subtherapeutic effects on sleep maintenance in the later portion of the night for some patients [5]. So, it is desired to maintain plasma concentration of drug for 8 hr.

Moreover, melt granulation is one of the most widely applied processing techniques in the array of pharmaceutical manufacturing operations due to its simplicity and easy scaleup [6–8]. In recent years, melt granulation has also been successfully employed to improve the dissolution rate of poorly soluble compounds increasing the bioavailability of these kinds of drugs, [9–11] and in the development of CR formulations [12–14] and masking the bitter taste of an active drug [15, 16]. 

Hence, the purpose of present investigation was to develope controlled-release tablet of Zolpidem tartrate by using polyethylene glycol (PEG 6000) [17] as melt binder, Hydroxypropyl methylcellulose (HPMC K4M) and Polyvinylpyrrolidone (PVP K30) as matrixing agent and filler, respectively, which would release the drug for prolonged period of time in view to maximize therapeutic effect of the drug and in an effort to expand the coverage of sleep complaints and overcome the lack of efficacy in sleep maintenance.

## 2. Materials and Methods

### 2.1. Materials

Zolpidem tartrate was procured from Tripada Pharmaceuticals Ltd., Ahmedabad, India. Hydroxypropyl methylcellulose K4M (HPMC K4M) was obtained from Yarrow Chem. Products, Mumbai, India. PEG 6000 and PVP K30 were obtained from S.D. Fine chemicals, Mumbai, India. Lactose, Magnesium stearate, and talc were purchased from Shakti Chemicals, Mehsana, India. All other materials and chemicals used were of either pharmaceutical or analytical grade.

### 2.2. Methods

#### 2.2.1. Preparation of Zolpidem Controlled-Release Tablets by Melt Granulation

Accurately weigh PEG 6000 was melted in a porcelain dish at 55–60°C on heating metal, and the accurate quantity of Zolpidem was added to the melted mass of PEG. Previously prepared geometric mixture by tumbling method using spatula for 14 minutes of HPMC K4M, PVP, and Lactose was added to the molten Zolpidem-PEG 6000 mixture and stirred well to mix. Then mass was removed from the hot plate and subjected to scrapping until it attained room temperature. The coherent mass was passed through 22 mesh, and the resulting granules were resifted over 44 meshes to separate granules and fines. The % loss of mass during melt granulation was found between 15 and 20% of total weight. The granules were collected and mixed with talc and magnesium stearate. The lubricated blend was compressed using 8 mm round flat punch on 10 station Rimek-I rotary tablet machine (Karnavati Engineering, Kadi, India). Compression was adjusted to obtain tablets with hardness in the range of 3-4 kg/cm^2^.

#### 2.2.2. Physical Characterization

The fabricated tablets were characterized for weight variation (*n* = 20), hardness (*n* = 6) Pfizer hardness tester (Janki Instrument Ltd, Ahmedabad, India), thickness using a screw-gauge micrometer (Campbell Electronics, Mumbai, India), and % friability (*n* = 20, Roche friabilator, Electrolab, Mumbai, India).

#### 2.2.3. *In Vitro* Dissolution Study

**Figure 1:**
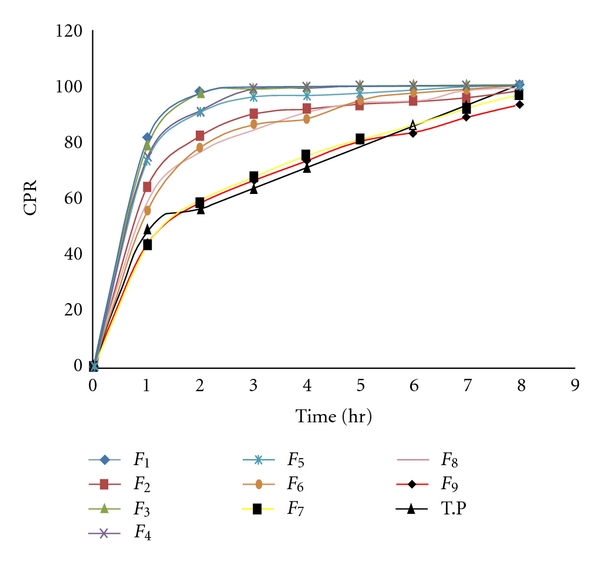
*In vitro* dissolution profile of factorial design batches.

The *in vitro* dissolution study of Zolpidem tablets (*n* = 3) was performed as described in Indian Pharmacopoeia 2010 using USP apparatus II (model TDT-08T, Electrolab, Mumbai, India) fitted with paddle (50 rpm) at 37°C ± 0.5°C using simulated gastric fluid (pH 1.2; 900 mL) as a dissolution medium for first 2 hours and followed by phosphate buffer (pH 6.8; 900 mL) for remaining hours. At the predetermined time intervals, 10-mL samples were withdrawn and analyzed at 238 nm using a Shimadzu UV 1800 double-beam spectrophotometer (Shimadzu, Kyoto, Japan). Cumulative percentage drug release was calculated using an equation obtained from a calibration curve which is developed in the range of 2–16 *μ*g/mL for 0.1 N HCl and pH-6.8 phosphate buffer (see Figure 1). 

#### 2.2.4. Optimization of Variables Using Full Factorial Design

A 3^2^ randomized full factorial design was employed in the present study. In this design, 2 factors were evaluated, each at 3 levels, and experimental trials were performed for all 9 possible combinations. The ratio of polymer (HPMC K4M : PVP) (*X*_1_) and concentration of melt binder (PEG 6000) (*X*_2_) were chosen as independent variables in 3^2^  full factorial design, while *Q*_1_, *Q*_4_, and *Q*_8_ (% drug release after 1, 4, and 8 hours, resp.), diffusion coefficient (*n*), and release rate constant (*K*) were taken as dependent variables. The composition of factorial design batches (*F*_1_ − *F*_9_) is shown in Table 1. The prepared formulations were evaluated for assay, friability, and hardness and *in vitro* release study. The results of evaluation parameters are shown in Table 2. Statistical treatment was carried out to the factorial design batches using design expert DX8 software.

#### 2.2.5. Kinetic Modeling of Dissolution Data

The dissolution profile of all batches was fitted to various models such as zero order, first order, Higuchi [18], Hixon and Crowell [19], and Korsmeyer et al. [20], to ascertain the kinetic of drug release. 

#### 2.2.6. Comparison of Dissolution Profiles for Selection of Optimum Batch

The similarity factor (*f*_2_) given by SUPAC guidelines for a modified release dosage form was used as a basis to compare dissolution profiles. The dissolution profiles are considered to be similar when *f*_2_ is between 50 and 100. The dissolution profile of products was compared using an *f*_2_ which is calculated from following formula:

(1) $$f_{2}=50\times \log\left\{{\left[1+\left(\frac{1}{n}\right)\overset{n}{\underset{t=1}{\sum }}w_{t}{\left(R_{t}-T_{t}\right)}^{2}\right]}^{-0.5}\times 100\right\},$$

where *n* is the dissolution time, and *R*_*t*_ and *T*_*t*_ are the reference (here, this is the theoretical dissolution profile of Zolpidem) and test dissolution value at time *t* [21].

#### 2.2.7. Fourier Transform Infrared Spectroscopy

Fourier transform infrared (FTIR) spectra of Zolpidem tartrate (see Figure 7) and granules of optimized batch were recorded using KBr mixing method on FTIR (FTIR-1700, Shimadzu, Kyoto, Japan) for drug excipients interaction study (see Figure 8).

## 3. Results and Discussion

### 3.1. Result of Preliminary Screening

From the in vitro dissolution study, it was found that hydrophobic binder MCC wax and bees wax have more sustaining effect on the release of drug than stearic acid and cetyl alcohol it is due to its hydrophobic nature. Hydrophilic binder PEG-6000 gave good drug release compared to all the other binders, which is due to its hydrophilic nature. HPMC K4M (hydrophilic) was selected as a matrixing agent considering its widespread applicability and excellent gelling activity in controlled-release formulations. PVP was also selected in formulation because it helps in releasing loading dose from the formulation in the 1st hour which is required for the therapeutic effect of formulation.

### 3.2. Full Factorial Design

A statistical model incorporating interactive and polynominal terms was used to evaluate the responses

(2) $$Y=b_{0}+b_{1}X_{1}+b_{2}X_{2}+b_{12}X_{1}X_{2}+b_{11}X_{1}^{2}+b_{22}X_{2}^{2},$$

where *Y* is the dependent variable, *b*_0_ is the arithmetic mean response of the 9 runs, and *b*_1_ is the estimated coefficient for the factor Xi. The main effects (*X*_1_ and *X*_2_) represent the average result of changing 1 factor at a time from its low to high values. The interaction terms (*X*_1_ *X*_2_) show how the response changes when two factors are simultaneously changed. The polynomial terms (*X*_1_^2^ and *X*_2_^2^) are included to investigate nonlinearity. The dissolution profile for 9 batches showed a variation (i.e., initial 1 hr release ranging from 43.22% to 81.33% and drug release after 8 hr ranging from 96.82% to 100%). The fitted equations (full and reduced) relating the responses, *Q*_1_, *Q*_4_, and *Q*_8_, diffusion coefficient (*n*), and release rate constant (*K*) to the transformed factor are shown in the Table 3. The polynomial equations can be used to draw conclusions after considering the magnitude of coefficient and the mathematical sign it carries (i.e., negative or positive).  Table 4 shows the results of analysis of variance (ANOVA), which was performed to identify insignificant factors. Data were analyzed using Design of Expert version 8.

*R* ^2^ values for *Q*_1_, *Q*_4_, diffusion coefficient (*n*), and release rate constant (*K*) are 0.7774, 0.7122, 0.8135, and 0.7867, respectively, indicating good correlation between dependent and independent variables. The low *R* ^2^ value, 0.6055 for *Q*_8_, indicates poor correlation between dependent and independent variables showing that drug release at 8 hr is less dependent on selected variables. The reduced models were developed for response variables by omitting the insignificant terms with *P* > .1000. The terms with *P* < .1000 were considered statistically significance and retained in the reduced model. The coefficients for full and reduced models for response variables are shown in Table 4. The significance levels of the coefficients in the *Q*_8_ were found to be insignificant at *P* > .1000 and, hence, do not contribute significant information to the prediction of *Q*_8_.

### 3.3. Full and Reduced Model for *Q*_1_

The significance levels of the coefficients *b*_1_, *b*_11_, *b*_22_, and *b*_12_ were found to be *P* = .4583,  .9722, .4441, and .9534, respectively, so they were omitted from the full model to generate a reduced model [22]. The results of statistical analysis are shown in Table 4. The coefficients *b*_0_  and  *b*_2_ were found to be significant at *P* < .1000; hence, they were retained in the reduced model. The reduced model was tested in proportion to determine whether the coefficients *b*_1_, *b*_11_, *b*_12_, and *b*_22_ contribute significant information to the prediction of *Q*_1_. The results of model testing are shown in Table 4. The critical value of *F* for *α* = 0.1 is equal to 5.34 (*df* = 4,3). Since the calculated value (*F* = 0.414) is less than the critical value (*F* = 5.34), it may be concluded that the interaction terms *b*_1_, *b*_11_, *b*_12_, and *b*_22_ do not contribute significantly to the prediction of *Q*_1_ and can be omitted from the full model to generate the reduced model.

### 3.4. Full and Reduced Model for *Q*_4_

The significance levels of the coefficients *b*_1_, *b*_11_, *b*_22_, and *b*_12_ were found to be *P* =  .6023,  .5762,  .390, and  .9344, respectively, so they were omitted from the full model to generate a reduced model. The results of statistical analysis are shown in Table 4. The coefficients *b*_0_, and *b*_2_ were found to be significant at *P* < .1000; hence, they were retained in the reduced model. The reduced model was tested in proportion to determine whether the coefficients *b*_1_, *b*_11_, *b*_12_, and *b*_22_ contribute significant information to the prediction of *Q*_4_. The results of model testing are shown in Table 4. The critical value of F for *α* = 0.1 is equal to 5.34 (*df* = 4,3). Since the calculated value (*F* = 0.4172) is less than the critical value (*F* = 5.34), it may be concluded that the interaction terms *b*_1_, *b*_11_, *b*_12_ and *b*_22_ do not contribute significantly to the prediction of *Q*_4_ and can be omitted from the full model to generate the reduced model.

### 3.5. Full and Reduced Model for Diffusion Coefficient (*n*)

The significance levels of the coefficients *b*_1_, *b*_11_, *b*_22_, and *b*_12_ were found to be *P* =  .4318,  .8711,  .4198,  and  1.000, respectively, so they were omitted from the full model to generate a reduced model. The results of statistical analysis are shown in Table 4. The coefficients *b*_0_ and *b*_2_ were found to be significant at *P* < .1000; hence, they were retained in the reduced model. The reduced model was tested in proportion to determine whether the coefficients *b*_1_, *b*_11_, *b*_12_, and *b*_22_ contribute significant information to the prediction of diffusion coefficient (*n*). The results of model testing are shown in Table 4. The critical value of *F* for *α* = 0.1 is equal to 5.34 (*df* = 4,3). Since the calculated value (*F* = 0.5717) is less than the critical value (*F* = 5.34), it may be concluded that the interaction terms *b*_1_, *b*_11_, *b*_12_, and *b*_22_ do not contribute significantly to the prediction of diffusion coefficient (*n*) and can be omitted from the full model to generate the reduced model.

### 3.6. Full and Reduced Model for Release Rate Constant (*K*)

**Figure 2:**
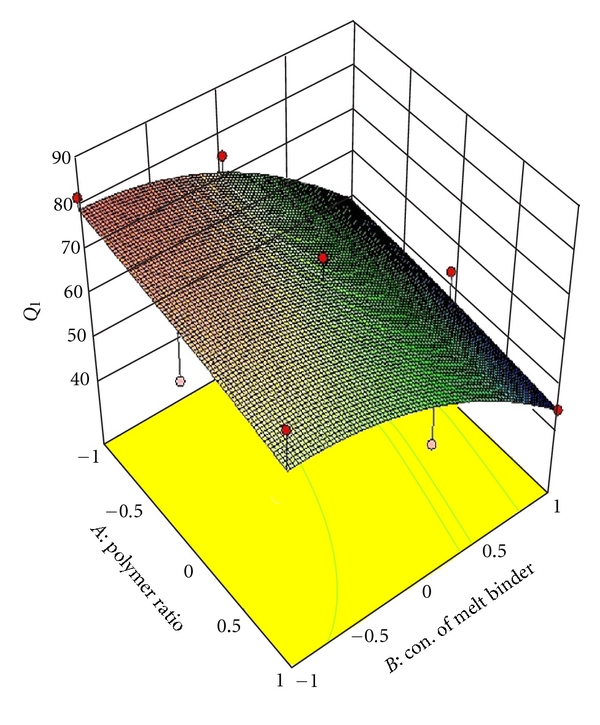
Response surface plot for *Q*_1_.

**Figure 3:**
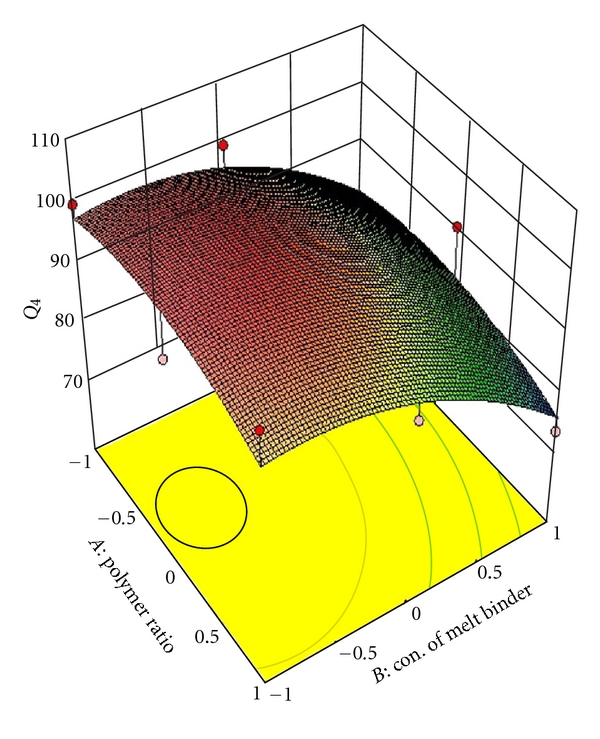
Response surface plot for *Q*_4_.

**Figure 4:**
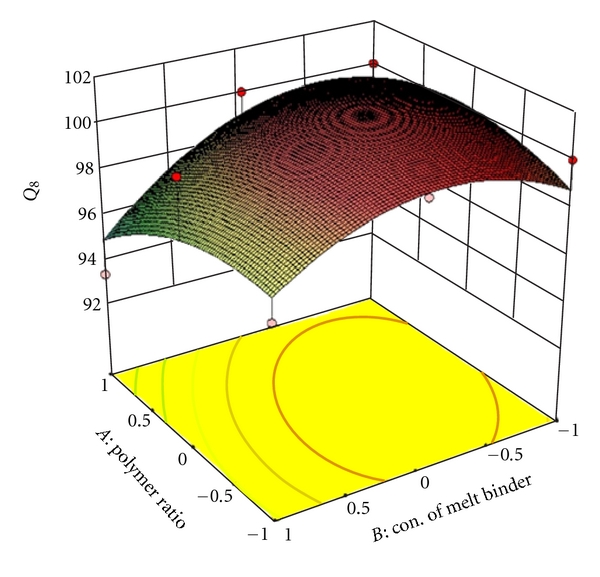
Response surface plot for *Q*_8_.

**Figure 5:**
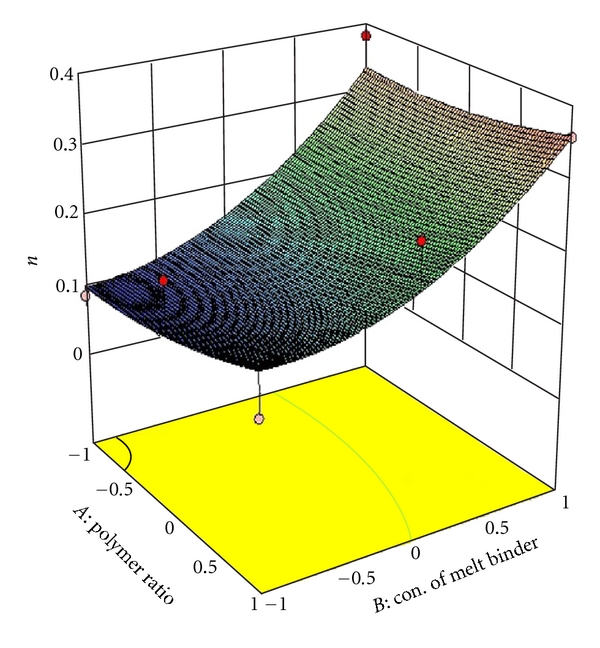
Response surface plot for diffusion coefficient (*n*).

**Figure 6:**
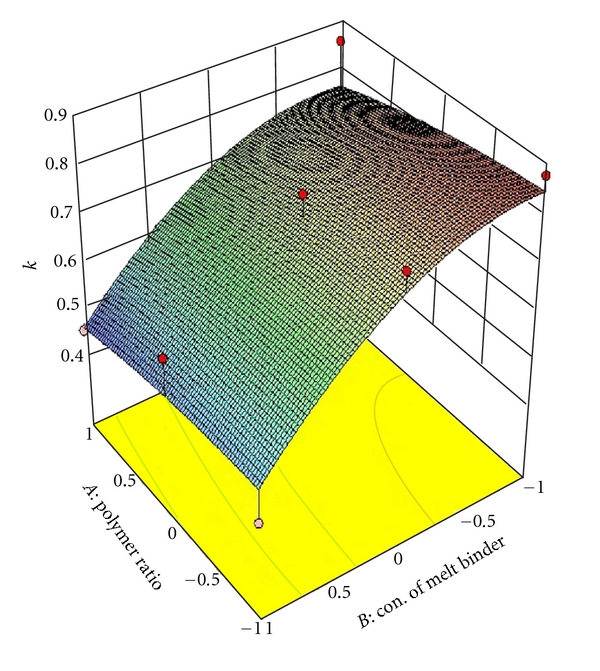
Response surface plot for release rate constant (*K*).

**Figure 7:**
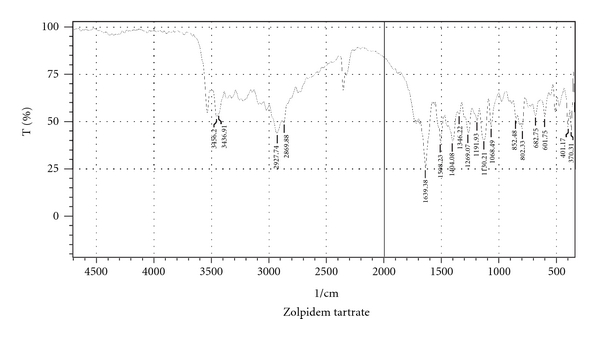
FTIR spectrum of Zolpidem tartrate.

**Figure 8:**
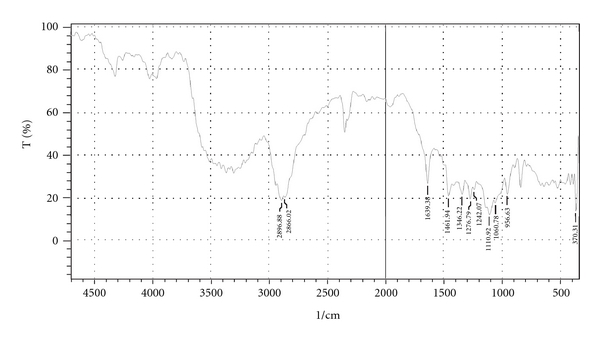
FTIR spectrum of granules of optimized batch.

The significance levels of the coefficients *b*_1_, *b*_11_, *b*_22_, and *b*_12_ were found to be *P* = .4880, .9164, .4095, and .9882, respectively, so they were omitted from the full model to generate a reduced model. The results of statistical analysis are shown in Table 4. The coefficients *b*_0_ and *b*_2_ were found to be significant at *P* < .1000; hence, they were retained in the reduced model. The reduced model was tested in proportion to determine whether the coefficients *b*_1_, *b*_11_, *b*_12_, and *b*_22_ contribute significant information to the prediction of release rate constant (*K*). The results of model testing are shown in Table 4. The critical value of *F* for *α* = 0.1 is equal to 5.34 (*df* = 4,3). Since the calculated value (*F* = 0.5717) is less than the critical value (*F* = 5.34), it may be concluded that the interaction terms *b*_1_, *b*_11_, *b*_12_, and *b*_22_ do not contribute significantly to the prediction of release rate constant (*K*) and can be omitted from the full model to generate the reduced model. To demonstrate graphically the effect of the ratio of polymer (HPMC K4M: PVP) and concentration of melt binder (PEG 6000), the response surface plots were generated by using Design expert 8.0.2 trial version software for the dependent variables Q_1_, Q_4_, Q_8_ (% drug release after 1, 4, 8 hours, resp.), diffusion coefficient (*n*), release rate constant (*K*) and shown in Figures 2, 3, 4, 5, and 6 respectively.

### 3.7. Kinetic Modeling of Dissolution Data

The kinetics of the dissolution data were well fitted to zero order, Higuchi model, and Krossmayer-Peppas model as evident from regression coefficients in Table 5. In case of the controlled-release formulations, diffusion, swelling, and erosion are the three most important rate controlling mechanisms. Formulation containing swelling polymers show swelling as well as diffusion mechanism because the kinetic of swelling includes relaxation of polymer chains and imbibitions of water, causing the polymer to swell and changing it from a glassy to rubbery state. The value of diffusion exponent *n* for most factorial formulations is between 0.084 and 0.379 (Table 5) indicating Fickian drug release from the formulations [23, 24].

### 3.8. Comparison of Dissolution Profiles for Selection of Optimum Batch

The values of similarity factor (*f*_2_) for the batch *F*_7_ showed maximum 72.22 (Table 2); hence, it was selected as optimum batch.

### 3.9. Fourier Transform Infrared Spectroscopy

The Zolpidem tartrate exhibits peak due to amide and alkenes group. It was observed that there were no changes in these main peaks in the FTIR spectra of a mixture of drug and polymers (Figure 3); hence, it was concluded that there were no physical or chemical interactions of Zolpidem with PEG 6000, PVP, and HPMC K4M. 

## 4. Conclusion

From the present investigation, it was concluded that the concentrations of PEG 6000 as a melt binder have more pronounced effect than the ratio of HPMC K4M and PVP K30 polymers on drug release from controlled-release tablet formulation and are useful to produced tablet dosage form with desirable drug release pattern.

## Figures and Tables

**Table 1:** Formulation and evaluation of batches in 3^2^ full factorial design.

Batch code	Variable levels in coded form		*Q*_1_	*Q*_4_	*Q*_8_	*n*	*K*
	*X*_1_	*X*_2_					
*F*_1_	−1	−1	81.33	99.67	100	0.084	0.868
*F*_2_	−1	0	63.91	91.76	98.29	0.188	0.686
*F*_3_	−1	1	78.73	99.6	100	0.097	0.848
*F*_4_	0	−1	74.91	99.19	100	0.128	0.802
*F*_5_	0	0	73.4	96.61	100	0.134	0.784
*F*_6_	0	1	55.58	88.14	100	0.268	0.603
*F*_7_	1	−1	43.22	75.26	96.82	0.379	0.44
*F*_8_	1	0	53.35	90.83	100	0.288	0.574
*F*_9_	1	1	43.9	73.32	93.33	0.356	0.446

Coded values	Actual values						
	*X*_1_	*X*_2_					

−1	25% : 20%	15%					
0	30% : 15%	20%					
1	35% : 10%	25%					

*All batches contained 12.5 milligrams of Zolpidem, 2.5 mg of talc, and 1.25 mg of magnesium stearate. *X*_1_ indicates the ratio of HPMC K4M (%): PVP (%), and *X*_2_ is the concentration of melt binder PEG 6000. *Q*_1_, *Q*_4_, and *Q*_8_ indicate the percentage of drug released after 1, 4, and 8 hours, respectively. *n* and *K* indicate diffusion coefficient and release rate constant, respectively.

**Table 2:** Results of factorial design batches (*F*_1_ − *F*_9_).

Parameter	*F*_1_	*F*_2_	*F*_3_	*F*_4_	*F*_5_	*F*_6_	*F*_7_	*F*_8_	*F*_9_
Assay (%)	93.45	97.43	92.50	91.45	94.62	96.08	95.2	92.74	97.6
Friability (%)	0.162	0.198	0.190	0.112	0.105	0.107	0.043	0.067	0.0982
Hardness (Kg/cm^2^)	3.25	3.5	3.0	2.75	3.75	4	3.75	3.25	3.75
Similarity factor (*f*_2_)	28.69	38.03	29.12	30.67	32.05	40.99	72.22	43.46	70.64

**Table 3:** Summary of the results of regression analysis.

*Q*_1_						
Response (*Q*_1_)	*b*_0_	*b*_1_	*b*_2_	*b*_12_	*b*_11_	*b*_22_
FM	68.16	−3.85	−13.60	0.3525	−0.296	−6.91
RM	63.14	—	−13.91	—	—	—

*Q*_4_						

Response (*Q*_6_)	*b*_0_	*b*_1_	*b*_2_	*b*_12_	*b*_11_	*b*_22_
FM	97.27	−2.11	−8.67	−0.367	−3.63	−6.30
RM	90.48	—	−8.60	—	—	—

*Q*_8_						

Response (*Q*_8_)	*b*_0_	*b*_1_	*b*_2_	*b*_12_	*b*_11_	*b*_22_
FM	101.06	−0.055	−1.88	−0.0825	−1.59	−2.45
RM	98.715		−1.35			

*n*						

Response (*n*)	*b*_0_	*b*_1_	*b*_2_	*b*_12_	*b*_11_	*b*_22_
FM	0.1704	0.0276	0.103	−0.00098	0.0093	0.049
RM	0.2135	—	0.109	—	—	—

*K*						

Response (*K*)	*b*_0_	*b*_1_	*b*_2_	*b*_12_	*b*_11_	*b*_22_
FM	0.7362	−0.039	−0.153	0.00095	−0.0098	−0.0823
RM	0.6723		−0.157			

FM = full model, RM = reduced model.

**Table 4:** Calculations for testing the model in portions.

	DF	SS	MS	F	R^2^	
*Q*_1_						
Regression						Fcalc. = 0.414
FM	5	1295.88	259.176	2.095	0.7774	Ftable = 5.34
RM	1	1162.04	1162.042	14.11	0.6684	DF(4,3)
Error						
FM	3	370.996	123.665			
RM	7	576.251	82.322			

*Q*_4_						

Regression						Fcalc. = 0.417
FM	5	588.807	117.76	1.48	0.7122	Ftable = 5.34
RM	1	444.104	444.104	8.39	0.5453	DF (4,3)
Error						
FM	3	237.936	79.31			
RM	7	370.317	52.9			

*Q*_8_						

Regression						Fcalc. = 0.197
FM	5	38.474	7.694813	0.9211	0.6055	Ftable = 5.34
RM	1	11.0432	11.0432	2.44	0.2587	DF (4,3)
Error			8.353			
FM	3	25.05915	4.5191			
RM	7	31.6339				

*n*						

Regression						Fcalc.= 0.571
FM	5	0.07328	0.0146	2.61	0.8135	Ftable=5.34
RM	1	0.07128	0.07128	16.88	0.7069	DF(4,3)
Error						
FM	3	0.01679	0.005597			
RM	7	0.02955	0.004222			

*K*						

Regression						Fcalc.=0.439
FM	5	0.1639	0.0327	2.21	0.7867	Ftable=5.34
RM	1	0.1478	0.1478	14.7	0.677	DF (4,3)
Error						
FM	3	0.0444	0.0148			
RM	7	0.0704	0.0101			

DF, degree of freedom; SS, sum of squares; MS, mean of squares; *R* ^2^, regression coefficient; FM, full model; RM, reduced model.

**Table 5:** Kinetic treatment of dissolution data.

	*F*_1_	*F*_2_	*F*_3_	*F*_4_	*F*_5_	*F*_6_	*F*_7_	*F*_8_	*F*_9_
Zero order									

*B*	1.7188	3.850	1.9727	2.6722	2.8542	5.42	7.1859	5.7625	6.6357
*A*	89.50	71.37	87.95	83.46	81.25	62.96	42.74	59.76	43.639
*R* ^2^	0.6497	0.8484	0.6567	0.7395	0.7897	0.8932	0.9799	0.9023	0.9775

First order									
*B*	0.0081	0.0203	0.0095	0.0130	0.0140	0.0296	0.0449	0.0321	0.0338
*A*	1.95	1.8528	1.9418	1.9192	1.9083	1.8012	1.6650	1.7806	1.7414
*R* ^2^	0.6438	0.8206	0.6482	0.7276	0.7699	0.8551	0.9483	0.8664	0.9394

Higuchi									
*B*	7.6048	16.077	8.710	11.572	69.396	22.35	28.5	23.64	26.37
*A*	81.73	55.93	79.075	71.90	12.119	41.81	16.90	37.51	19.75
*R* ^2^	0.7375	0.9088	0.7429	0.8216	0.8602	0.9439	0.9974	0.9497	0.9962

Hixon Crowell									
*B*	−0.028	−0.0678	−0.0327	−0.044	−0.0480	−0.0976	−0.141	−0.105	−0.131
*A*	0.1726	0.4946	0.1998	0.2764	0.3136	0.6578	1.0844	0.7219	1.069
*R* ^2^	−0.645	−0.8300	−0.6507	−0.7316	−0.7765	−0.8684	−0.9605	−0.8792	−0.9594

Korsmeyer and Peppas									
*B*	0.0837	0.187	0.097	0.128	0.133	0.267	0.379	0.288	0.356
*A*	−0.0621	−0.163	−0.0711	−0.095	−0.105	−0.219	−0.355	−0.240	−0.350
*R* ^2^	0.8201	0.9435	0.8229	0.8886	0.9109	0.962	0.998	0.968	0.997

*B* = slope, *A* = intercept, *R* ^2^= square of correlation coefficient, and *n*= diffusion exponent.
